# Supplementary material for: Structure of the conjugation surface exclusion protein TraT
Source: Commun Biol. 2025 Nov 26;8:1702. doi: 10.1038/s42003-025-09102-8 (PMC12658005; doi:10.1038/s42003-025-09102-8)
Supplement: Supplementary file 1 — Supplementary Information [file 42003_2025_9102_MOESM1_ESM.pdf]

Supplementary Information

Structure of the conjugation surface exclusion protein TraT

Nicolas Chen[1], Alfredas Bukys[1,3,4 †], Camilla A.K. Lundgren[1†], Justin C. Deme[2], Hafez El Sayyed[3,4], Achillefs N. Kapanidis[3,4], Susan M. Lea[2,5*], Ben C. Berks[1*].

[1]Department of Biochemistry, University of Oxford, Oxford, OX1 3QU, United Kingdom.

[2]Center for Structural Biology, Center for Cancer Research, National Cancer Institute, Frederick, MD 21702, United States of America.

[3]Biological Physics Research Group, Department of Physics, University of Oxford, Oxford OX1 3PU, United Kingdom.

[4]Kavli Institute for Nanoscience Discovery, University of Oxford, Sherrington Road, Oxford OX1 3QU, United Kingdom.

[5]Structural Biology, St Jude Children's Research Hospital, Memphis, Tennessee, United States of America.

[†] These authors contributed equally to this work.

*Correspondence to: ben.berks@bioch.ox.ac.uk, susan.lea@stjude.org

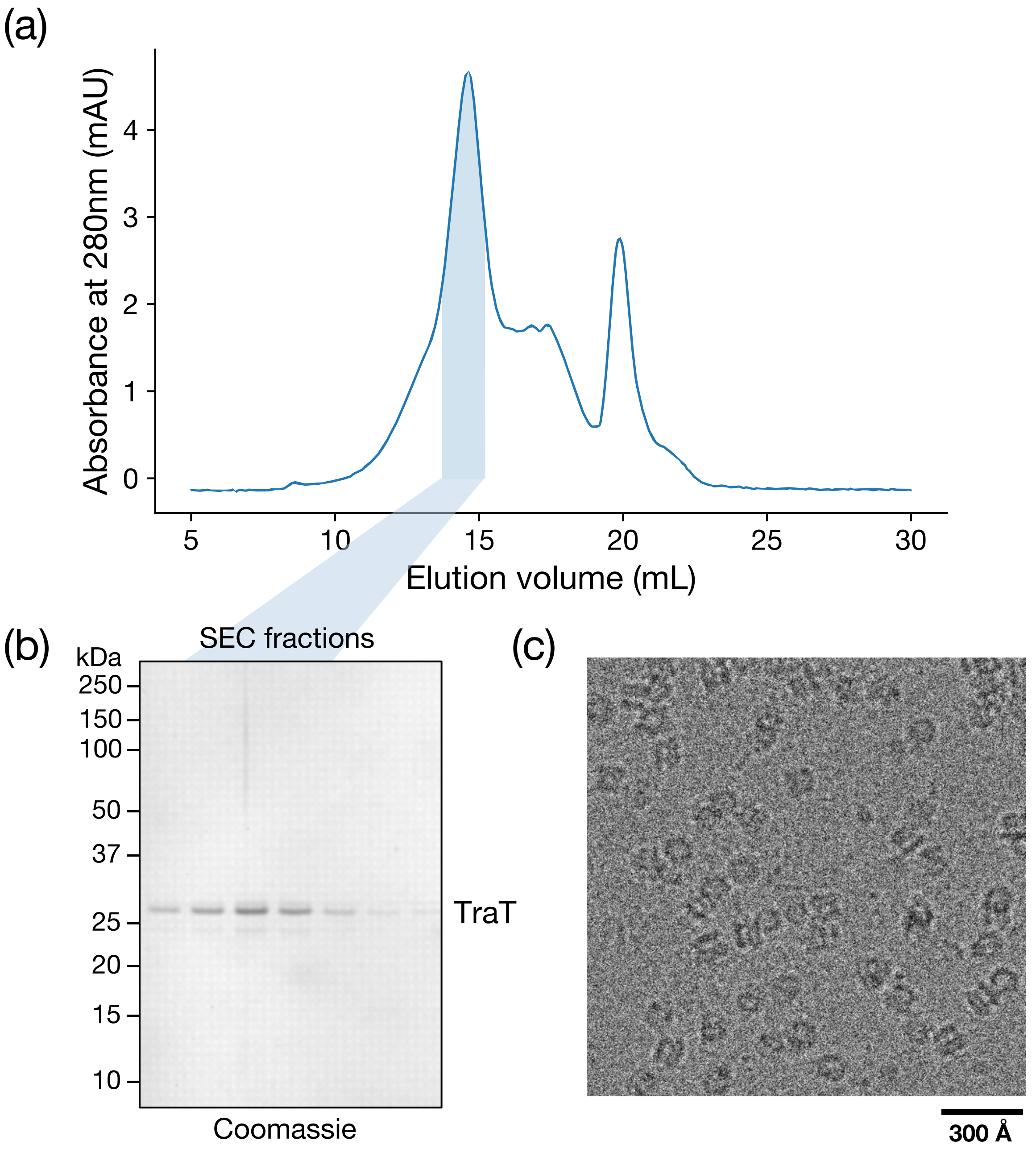

Supplementary Figure 1. Purification of the F plasmid TraT complex.

Twin-Strep tagged recombinant TraT was solubilized from membranes using DDM, and isolated by streptactin affinity chromatography followed by SEC.

a,b SEC elution profile **(a)** and Coomassie stained SDS-PAGE gel **(b)** of the SEC peak fractions.

c, Cryo-EM image of the pooled fractions shown shaded in blue in (a,b).

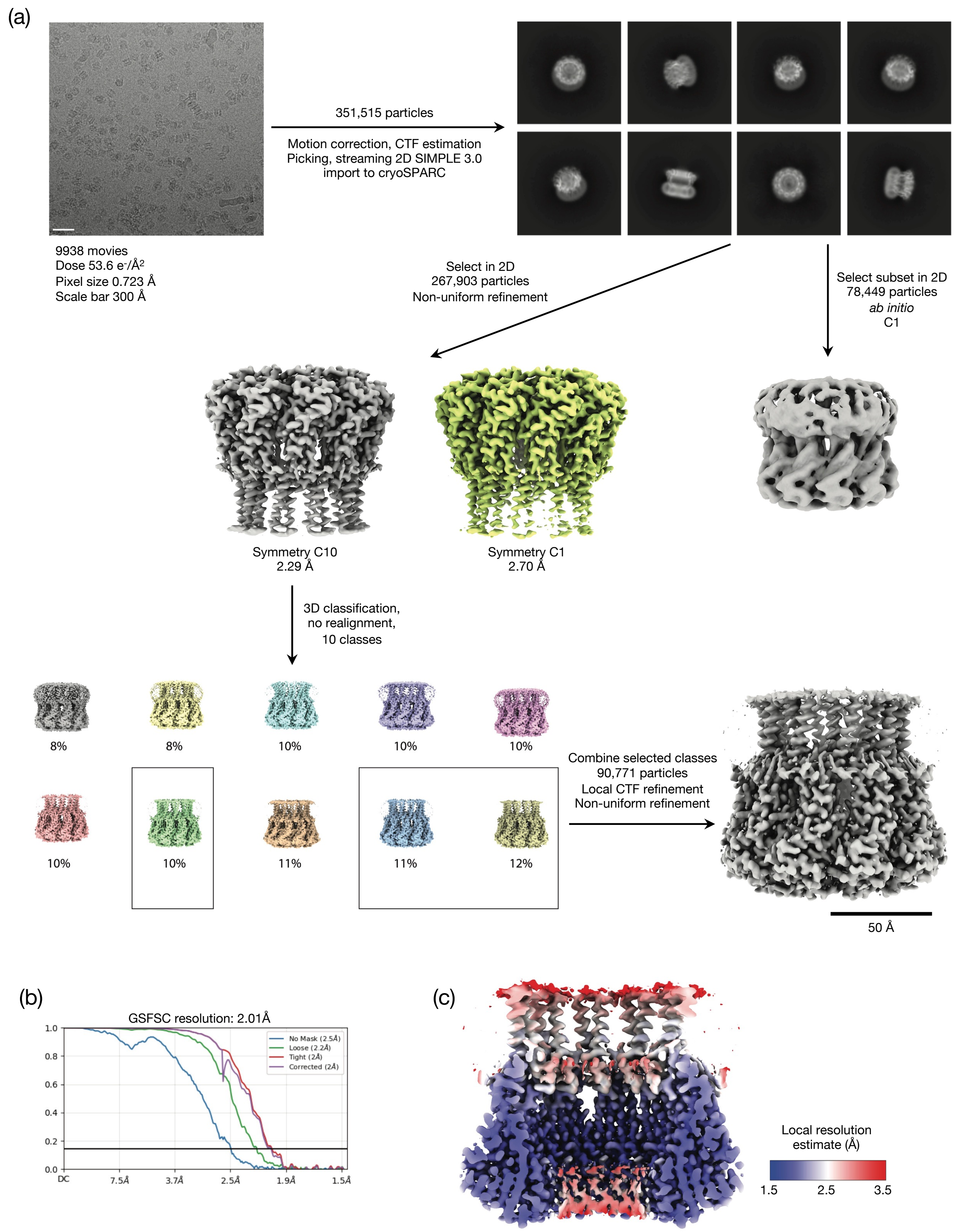

Supplementary Figure 2. Cryo-EM workflow for the F plasmid TraT complex.

a, Image processing workflow, Micrograph scale bar is 300 Å.

b, Gold-standard Fourier Shell Correlation (FSC) curves used for global resolution estimation.

c, Local resolution estimate of the volume.

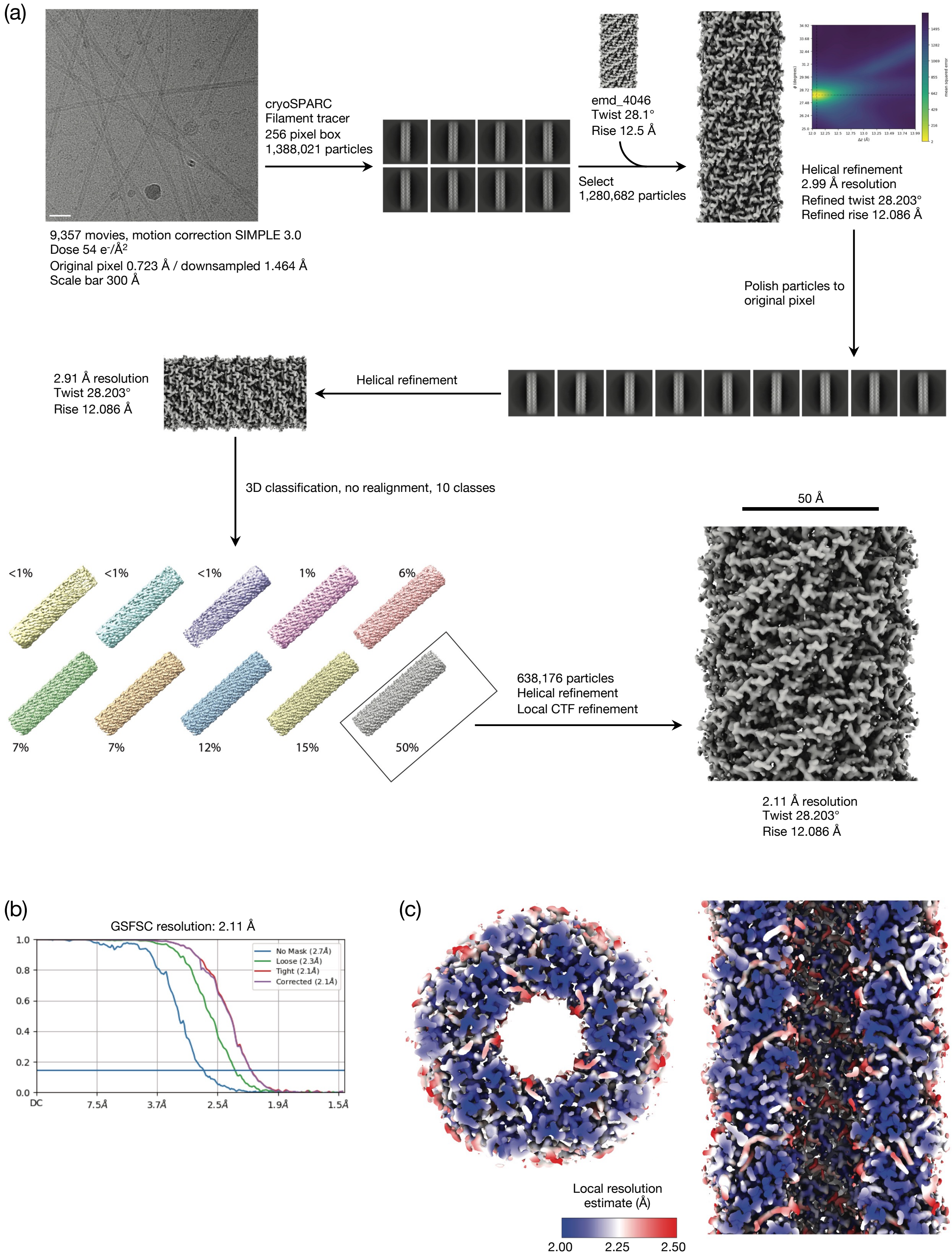

Supplementary Figure 3. Cryo-EM workflow for the pED208 pilus.

a, Image processing workflow, Micrograph scale bar is 300 Å.

b, Gold-standard Fourier Shell Correlation (FSC) curves used for global resolution estimation.

c, Local resolution estimate of the volume.

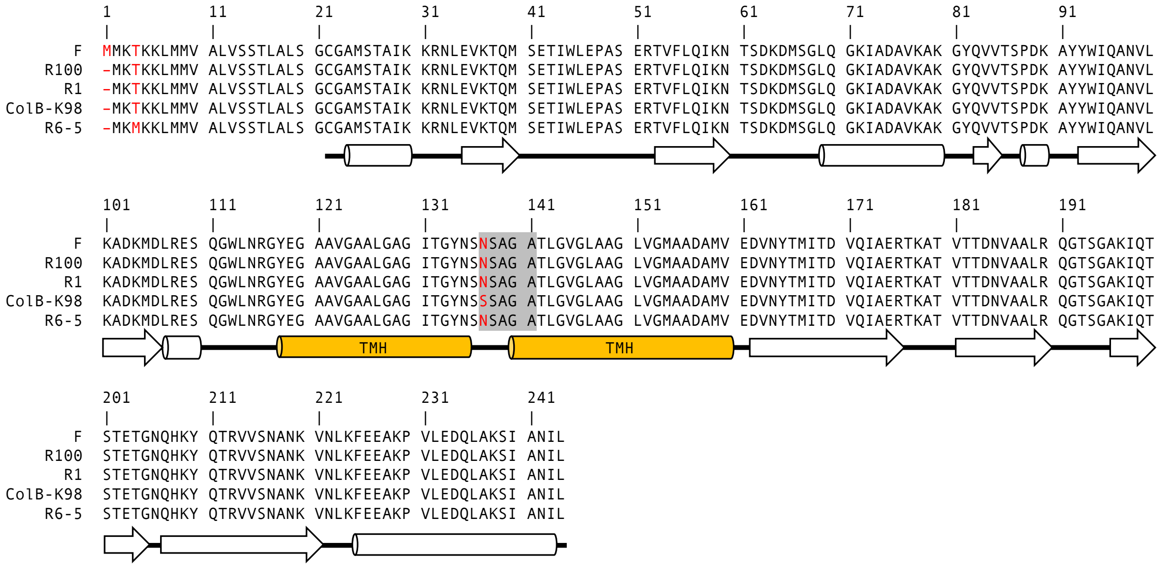

Supplementary Figure 4. Sequence alignment of F TraT with close homologues encoded by other IncF plasmids. Sequence differences are indicated in red. The shaded region indicates the specificity region proposed in earlier work. The secondary structure diagram is plotted according to the cryo-EM structure of TraT (cylinders for α helices, arrows for β sheets). Orange cylinders represent transmembrane helices (TMH). GenBank accession numbers for the protein sequences from different plasmids are: BAA97971.1 (F), WP_000850422.1 (R100), KY749247.1 (R1), CAA32704.1 (ColB-K98), CAA36788.1 (R6-5).

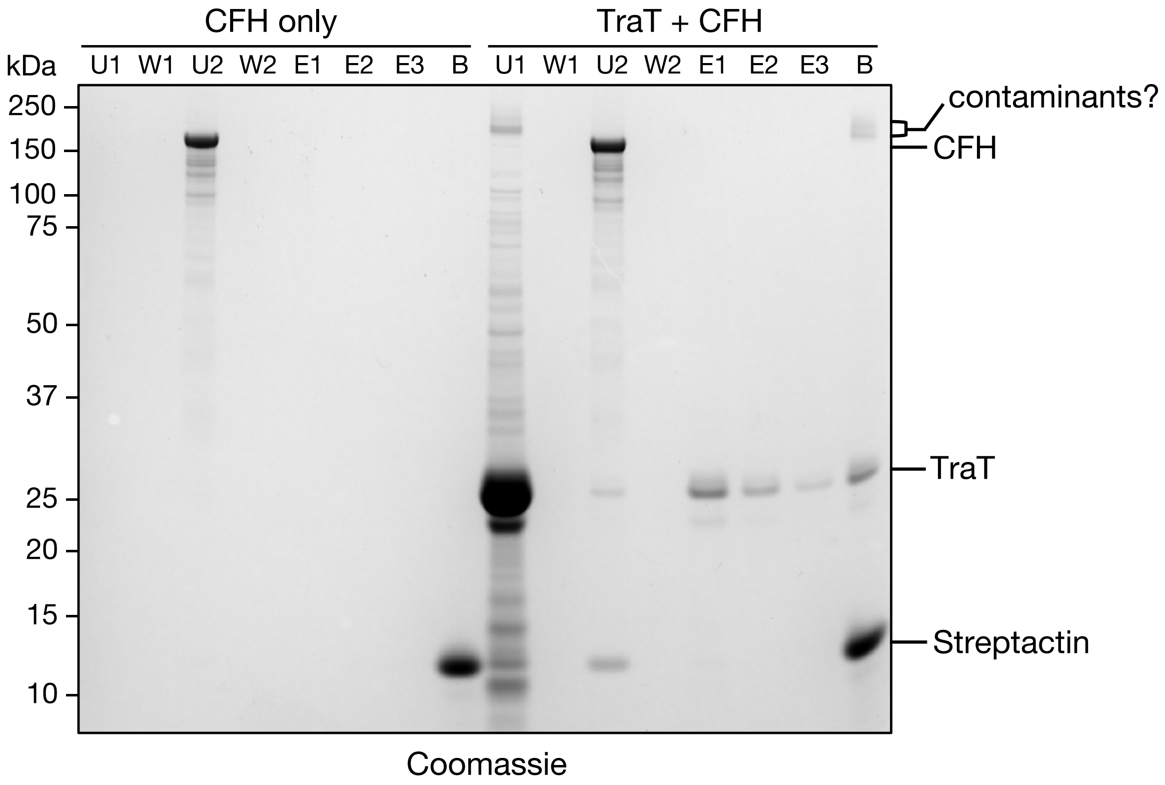

Supplementary Figure 5. TraT fails to interact with CFH.

MagStrep beads were incubated with a saturating level of purified TraT-TS (`TraT + CFH' lanes) or with a buffer blank (`CFH only' lanes). The beads were then recovered (U1 is the residual unbound fraction), washed (W1 is the final wash fraction), and incubated with mouse CFH. The beads were then recovered (U2 is the residual unbound fraction), washed (W2 is the final wash fraction), subjected to elution with desthiobiotin (elution fractions E1-E3), before being boiled at 100°C for 5 min in Laemmli buffer (B sample). Fractions were analyzed on a Coomassie Blue stained SDS-PAGE gel.

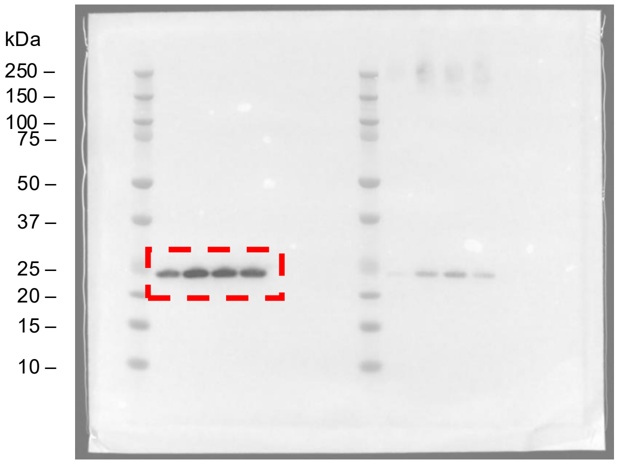

Fig. 4b (anti-TraT)

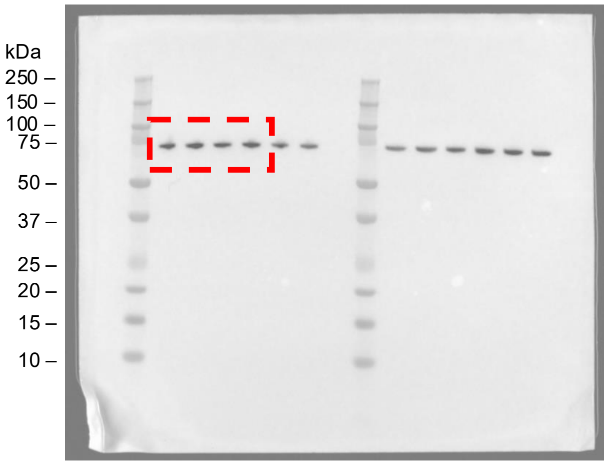

Fig. 4b (anti-DnaK)

Supplementary Figure 6. Source data for Fig. 4a,b.

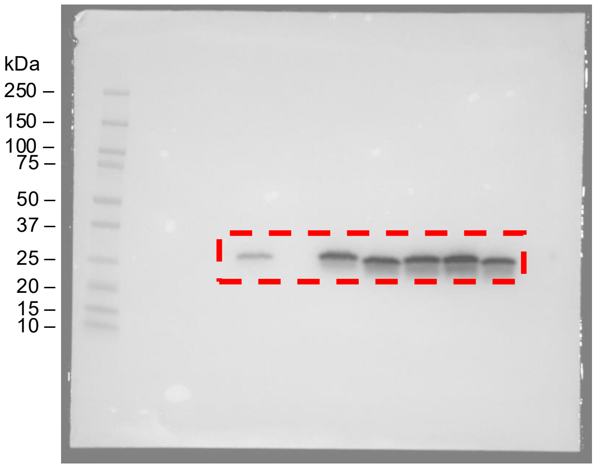

Fig. 5c (anti-TraT)

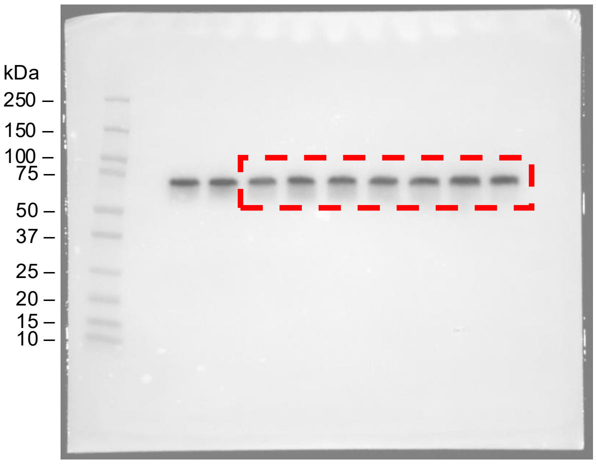

Fig. 5c (anti-DnaK)

Supplementary Figure 7. Source data for Fig. 5b,c.

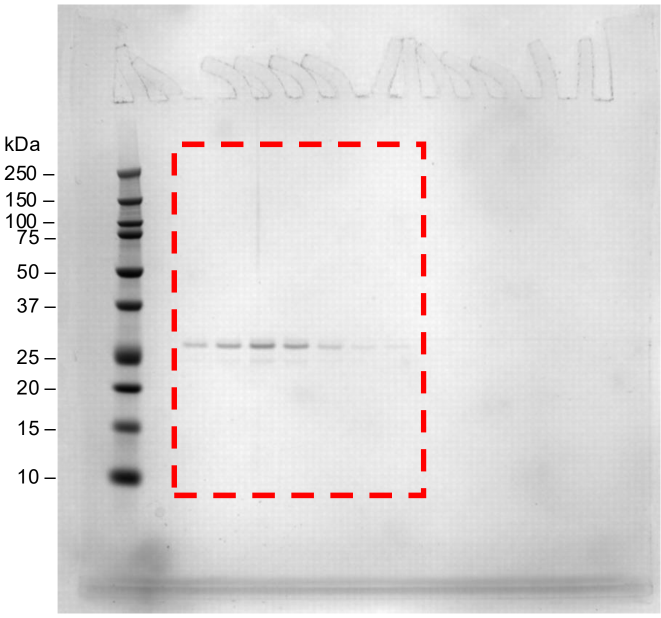

Supplementary Fig. 1b

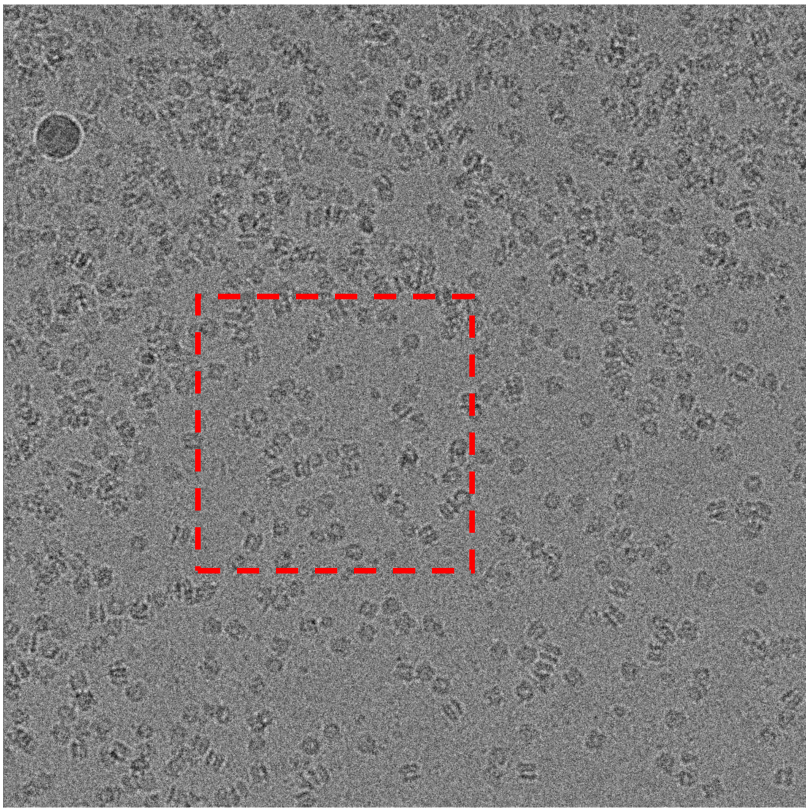

Supplementary Fig. 1c

Supplementary Figure 8. Source data for Supplementary Fig. 1b,c.

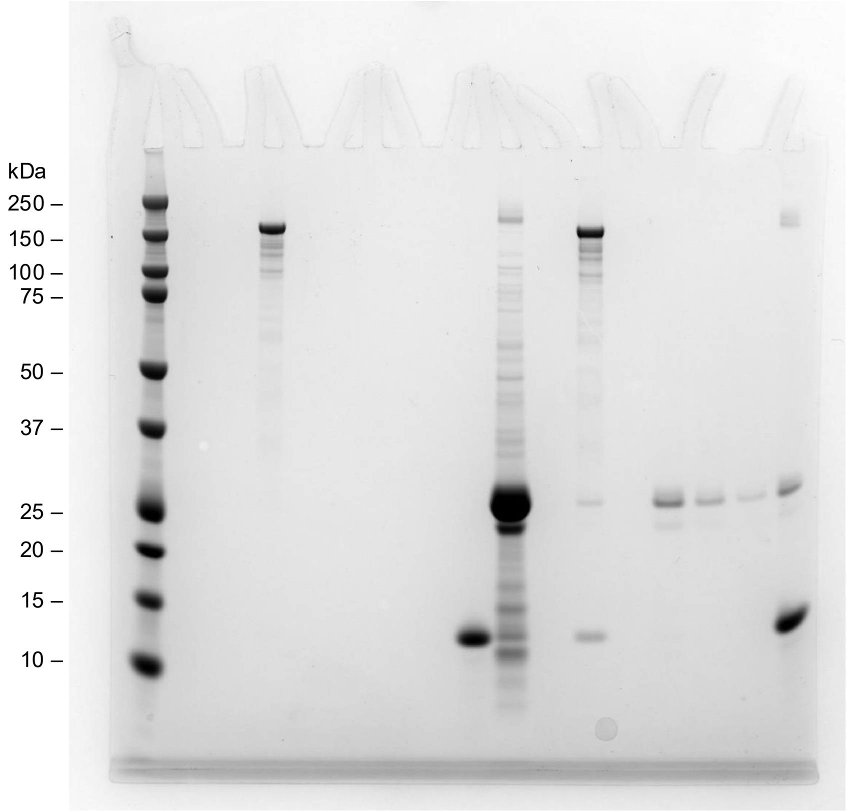

Supplementary Fig. 5

Supplementary Figure 8. Source data for Supplementary Fig. 5.

Supplementary Table 1. Strains and plasmids used in this study

E. coli strain	Genotype	Description	Source
MG1655	*F^- λ^- ilvG$^-$ rfb-50 rph-1*		[1]
MG_mSC	MG1655*mScarlet-I::Tn7*	Produces mScarlet-I	[2]
AB_2	*MG1655 ΔlacI ΔlacZ p^{rpoN}-tetR-mNeonGreen*	Produces TetR-mNeonGreen	This study
C41(DE3)	*F^- ompT gal dcm hsdS$_B$(r$_B^-$ m$_B^-$)(DE3)*	Overexpression strain	[3]
MC4100-AN	*F^- araD139 Δ(argF-lac)U169 rpsL150 (Str^R)relA1 flbB5301 deoC1 ptsF25 rbsR Ara^R Nal^R*	Has spontaneous arabinose and nalidixic acid resistance mutations	[4] and this study

Plasmid	Details/insert	Reference
pOX38-Kan^R	Largest HindIII fragment of the F plasmid circularised to incorporate a Kan^R deterimant Contains all *tra* and maintenance genes.	[5]
pOX38$_{tetOx22}$ (F$_{tetO}$)	pOX38-Kan^R with 22 *tetO* repeats inserted between the *slt* and *ygeB genes*	This study
pED208	Derepressed conjugative plasmid derived from F$_0$*lac*. Overpiliated phenotype.	[6]
pKD3	Contains the FRT-Cam^R-FRT cassette. Rep101 backbone	[7]
pKD3_tetOx22	pKD3 with 22 *tetO* sites	This study
pCP20	Encodes the FLP recombinase to remove Cam^R cassette used in λ red recombination. Temperature sensitive.	[7]
pKD46	Encodes the λ red-gam system used to insert fragments via λ red recombination. Temperature sensitive origin. Cam^R.	[7]
pROD51	*tetR-YPet*	Rodrigo Reyes-Lamothe

pLAU44	240x*tetO* repeats	[8]
pHAF-mNG	*mNeonGreen*	[9]
pTOF24	Scarless recombineering cloning vector	[10]
pAB1	*rpoN* promoter	This study
pAB2	Specifies a TetR-11aa linker-mNeonGreen fusion	This study
pAB3	pAB1 *tetR-mNeonGreen*	This study
pAB4	pTOF24 containing homology arms to *lacZ* and *mphR.*	This study
pAB5	pAB4 p^{rpoN}*TetR-mNeonGreen*	This study
pET22b-ts	pET22b vector fusing a TwinStrep tag coding sequence to the end of the inserted gene.	Berks lab
pNC60	pET22b-ts with *traT* insert	This study
pQE80L	IPTG-inducible expression plasmid.	Qiagen
pNC56 (pTraT-NSAGA)	pQE80L *traT*	This study
pNC91 (pTraT-NSAGG)	pQE80L $traT(_{137}NSAGG_{141})$	This study
pNC92 (pTraT-SSAGA)	pQE80L $traT(_{137}SSAGG_{141})$	This study
pNC99 (pTraT-D170A)	pQE80L $traT_{D170A}$	This study
pNC100 (pTraT-R213A)	pQE80L $traT_{R213A}$	This study
pNC101 (pTraT-R213E)	pQE80L $traT_{R213E}$	This study
pNC102 (pTraT-D170A-R213A)	pQE80L $traT_{D170A,R213A}$	This study

Supplementary References

1. Guyer M, Reed R, Steitz J, Low K. Identification of a sex-factor-affinity site in *E. coli* as γδ. In: *Cold Spring Harbor symposia on quantitative biology* **45**, 135-40). Cold Spring Harbor Laboratory Press (1981).

2. Granato ET, Smith WPJ, Foster KR. Collective protection against the type VI secretion system in bacteria. *The ISME Journal* **17**, 1052-1062 (2023).

3. Miroux B, Walker JE. Over-production of Proteins in *Escherichia coli*: Mutant hosts that allow synthesis of some membrane proteins and globular proteins at high levels. *Journal of Molecular Biology* **260**, 289-298 (1996).

4. Ize B*, et al. In vivo* dissection of the Tat translocation pathway in *Escherichia coli. Journal of Molecular Biology* **317**, 327-335 (2002).

5. Chandler M, Galas DJ. Cointegrate formation mediated by Tn*9*: II. Activity of IS*1* is modulated by external DNA sequences. *Journal of molecular biology* **170**, 61-91 (1983).

6. Falkow S, Baron LS. Episomic element in a strain of *Salmonella typhosa*. *Journal of Bacteriology* **84**, 581-589 (1962).

7. Datsenko KA, Wanner BL. One-step inactivation of chromosomal genes in *Escherichia coli* K-12 using PCR products. *Proceedings of the National Academy of Sciences* **97**, 6640-6645 (2000).

8. Lau IF, Filipe SR, Søballe B, Økstad O-A, Barre F-X, Sherratt DJ. Spatial and temporal organization of replicating *Escherichia coli* chromosomes. *Molecular Microbiology* **49**, 731-743 (2003).

9. El Sayyed H, Pambos OJ, Stracy M, Gottesman ME, Kapanidis AN. Single-molecule tracking reveals the functional allocation, *in vivo* interactions, and spatial organization of universal transcription factor NusG. *Molecular Cell* **84**, 926-937.e924 (2024).

10. Merlin C, McAteer S, Masters M. Tools for characterization of *Escherichia coli* genes of unknown function. *J Bacteriol* **184**, 4573-4581 (2002).
