# Supplementary material for: Structure of the conjugation surface exclusion protein TraT
Source: Commun Biol. 2025 Nov 26;8:1702. doi: 10.1038/s42003-025-09102-8 (PMC12658005; doi:10.1038/s42003-025-09102-8)
Supplement: Supplementary file 2 — Description of Additional Supplementary Files [file 42003_2025_9102_MOESM2_ESM.pdf]

Description of additional supplementary files-

File name: Supplementary Data 1.

Description: List of primers.

File name: Supplementary Data 2.

Description: Source Data for Fig. 4a.

File name: Supplementary Data 3.

Description: Source Data for Fig. 5b.
